# The complete mitochondrial genome of wild mung bean (*Vigna radiata* var. *sublobata* TC1966)

**DOI:** 10.1080/23802359.2019.1664953

**Published:** 2019-09-16

**Authors:** Ching-Ping Lin, Roland Schafleitner, Chien-Yu Chen, Hsiao-Feng Lo, Long-Fang Oliver Chen

**Affiliations:** a Institute of Plant and Microbial Biology, Academia Sinica, Taipei, Taiwan;; b Department of Earth and Life Sciences, University of Taipei, Taipei, Taiwan;; c Biotechnology/Molecular Breeding, World Vegetable Center, Tainan, Taiwan;; d Department of Bio-Industrial Mechatronics Engineering, National Taiwan University, Taipei, Taiwan;; e Department of Horticulture and Landscape Architecture, National Taiwan University, Taipei, Taiwan

**Keywords:** Wild mung bean, mitochondrial genome, *Vigna radiata* var. *sublobata*, domestication

## Abstract

The entire mitogenome of wild mung bean (*Vigna radiata* var. *sublobata* TC1966) was identified as a circular molecule of 402,981 bp length. The wild mung bean mitogenome encoded 3 rRNAs, 16 tRNAs, and 33 proteins. A phylogenetic tree was reconstructed using the 18 protein-coding genes of 14 legumes and one close species, *Ricinus communis*. Our phylogenetic analysis suggests that the wild mung bean clustered with the *Vigna radiata* var. *radiata*, as well as, the species of *Vigna* and *Glycine* appeared as a monophyletic group. This complete mitogenome sequence provides a genomic resource for further studies in mung bean breeding and domestication.

The seeds and sprouts of mung bean (*Vigna radiata* var. *radiata*) not only contain abundant nutrients but also potentially have health benefits, such as antidiabetic, antihypertensive, lipid metabolism accommodation, antihypertensive, and antitumor effects, etc (Tang et al. 2014). Mung bean has been widely used as a common food in China, India, and southeast Asian countries, for more than 2000 years. Mung bean has been domesticated and cultured by human beings for a long time. The putative progenitor of mung bean is *Vigna radiata* var. *sublobata* (Roxb.) Vercourt, which is distributed across the old world tropics from western Africa to northern Australia and Papua New Guinea (Tomooka et al. 2002). Researchers commonly use the variety *sublobata* (wild mung bean) as reference to study the breeding and domestication issues of mung bean. To date, several entire mitochondrial genomes of mung bean cultivars, such as Berken (Alverson et al. 2011) and NM92 (Lin et al. 2015), were reported, but that of wild mung bean is lacking. Here, we complete an entire mitochondrial genome sequence of wild mung bean to provide genomic resource for further researches.

The seed of wild mung bean (*V*. *radiata* var. *sublobata* TC1966) was obtained from AVRDC – The World Vegetable Center, Taiwan, and then was germinated and planted in the greenhouse of the Institute of Plant and Microbial Biology, Academia Sinica, Taiwan. The extraction and sequencing of wild mung bean genomes were reported in our previous study (Lin et al. 2015). The 29.17 million clean Illumina paired-reads were employed for assembling the mitogenome of wild mung bean using MITObim v1.7 (Hahn et al. 2013), with that of the congener cultivar NM92 (accession number: AP014716) (Lin et al. 2016) as the initial reference. The mitogenome sequence of wild mung bean has been annotated by BLAST searches (http://blast.ncbi.nlm.nih.gov/Blast.cgi). All tRNA genes were verified using tRNAscan-SE 1.21 (Schattner et al. 2005). The mitogenome sequence of wild mung bean was submitted to DDBJ with the accession number of AP014717. The mitogenome has a circular structure with 402,981 bp in length that is larger than its congener cultivar NM92 due to the expansion of the intronic regions. The contents of A, T, C, and G in the wild mung bean mitogenome were found to be 27.45, 27.45, 22.58, and 22.52%, respectively. The wild mung bean mitogenome encoded 3 rRNAs, 16 tRNAs, and 33 proteins.

The DNA sequences of 18 common protein-coding genes from 14 legumes were concatenated for phylogenetic analysis. A close species, *Ricinus communis*, was selected as the outgroup. The best-fit, GTR + G + I, nucleotide substitution model was employed in plant mitogenome phylogenetic reconstruction. A maximum-likelihood tree was reconstructed using MEGA7 program (Kumar et al. 2016) with 1000 bootstrap replicates. The wild mung bean clustered with the *V*. *radiata* var. *radiata*, as well as, the species of *Vigna* and *Glycine* appeared a monophyletic group (Figure 1). Our study would provide useful genomic resource for further studies in mung bean breeding and domestication.

**Figure 1.**
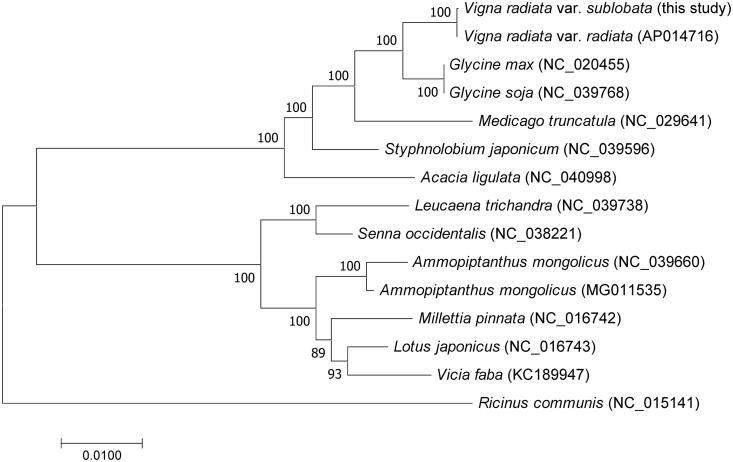
The mitogenome phylogenetics of wild mung bean and other legumes. A maximum-likelihood tree inferred from analysis of a data set containing 18 concatenated protein-coding genes in 15 mitogenomic taxa by use of the GTR + I + G model. Numbers at each node indicate bootstrap support. GenBank accession numbers of the species used in this phylogenetic tree are enclosed in brackets.
